# Liver-related mortality in homeless-experienced adults over a 16-year period

**DOI:** 10.21203/rs.3.rs-5417681/v1

**Published:** 2024-11-27

**Authors:** Logan Adams, Kirsten Dickins, Elizabeth Lewis, Marguerite Beiser, Travis Baggett, Danielle Fine

**Affiliations:** McLeod Centers for Wellbeing; Rush University - College of Nursing; Boston Health Care for the Homeless Program; Boston Health Care for the Homeless Program; Massachusetts General Hospital; Massachusetts General Hospital

## Abstract

Homeless-experienced adults have higher liver-related mortality than the general population. The objective of our study was to examine temporal liver-related mortality trends and assess cause-specific liver-related mortality disparities in a large cohort of homeless-experienced adults. We linked a cohort of 60,092 adults who received care at Boston Health Care for the Homeless Program (BHCHP) from 2003–2017 to Massachusetts death occurrence files spanning 2003–2018. We evaluated temporal trends in age-standardized liver-related mortality rates in comparison to the MA population. We identified the leading causes of liver-related death aggregated across the study period and compared these cause-specific mortality rates to the general population, reporting standardized mortality rate ratios (SRRs). Of the 7,130 deaths in the cohort, 652 (9.1%) were liver-related. Among liver-related decedents, the mean age at death was 55.7 years (SD 8.3); 517 (79.2%) were male and 399 (61.2%) were White. Liver-related mortality decreased on average 3.5% annually (95% CI: −6.6%, −0.3%), though remained significantly higher than the MA population throughout the study period. Leading causes of liver-related death were cirrhosis (n=157, SRR 3.2 [95% CI: 3.2, 3.3]), liver cancer (n=148, SRR 2.4 [95% CI: 2.4, 2.5]), alcohol-related liver disease (n=140, SRR 4.4 [95% CI: 4.3, 4.6]), and viral hepatitis (n=99, SRR 7.2 [95% CI: 6.9, 7.6]).Liver-related deaths are an important contributor to excess mortality among homeless-experienced adults. Efforts to reduce this disparity should address alcohol use disorder and viral hepatitis given their substantial contribution to the disparity of mortality in this population.

## Introduction

The all-cause mortality disparity between homeless-experienced adults and the general population is striking. Since the 1980s, mortality has been consistently described as 2- to 4-fold higher in homeless-experienced populations, and recent evidence suggests this disparity is widening.^1^ The reasons for this are complex; a number of “upstream” structural and social factors – extreme poverty, trauma, racism, poor nutrition, and competing priorities for survival – may lead to “downstream” causes of poorer health and higher mortality in this population.^2^

Liver-related mortality is a leading cause of death in homeless-experienced adults^1^ with liver-related mortality rates ranging 2- to 3-fold higher than in the general population.^3,4^ Liver-related mortality is a comprehensive cause of death category that encompasses various causes (i.e., viral hepatitis, autoimmune, and alcohol-related) and sequelae (i.e., cirrhosis, hepatic encephalopathy, and hepatorenal syndrome) of liver disease. ^5–7^ Despite the high prevalence of liver-related death in homeless-experienced adults, studies focusing on this broad categorization of death in this population are limited and, notably, do not parse out the subcategories of liver-related death.

In the U.S. general population, underlying causes of liver-related mortality have shifted over time. Between 2007 and 2016, mortality attributable to alcohol-related liver disease (ALD) and non-alcohol related liver disease (NAFLD) rose annually by 3.3% and 12.3%, respectively, while mortality attributable to hepatitis B virus (HBV) and hepatitis C virus (HCV) declined by 5.6% and 3.6% annually.^5^ ALD mortality, in particular, has accelerated in recent years, which may be related to the COVID-19 pandemic.^6^ Since the introduction of direct acting antivirals (DAAs) in 2013, HCV mortality has declined rapidly.^7^ By demographics, cirrhosis mortality is increasing more rapidly in females than males (2.9% vs 2.0% per year) and in non-Hispanic White individuals than Hispanic and Black individuals (2.9% vs. 1.0% vs. 0.9% per year, respectively).^5^

Understanding the trends in and subcategories of liver-related mortality in homeless-experienced adults is critical for the development of effective interventions to reduce health disparities in this population. Therefore, the objective of our study was to perform a comprehensive evaluation of liver-related mortality in a large cohort of people experiencing homelessness. Specifically, we aimed to (1) assess overall liver-related mortality by demographic subgroup and over time and (2) examine the subcategories of liver-related mortality and compare these cause-specific rates to those in the general population.

## Methods

### Participants and Setting

We used data from BHCHP’s electronic health record to create a cohort of adults ≥ 18 years of age who had at least one in-person encounter at the Boston Health Care for the Homeless Program (BHCHP) between January 1, 2003 and December 31, 2017. The study entry date was the date of the first encounter at BHCHP that occurred during the study period. The study exit date was either (1) the date of death for patients who died during the observation period or (2) December 31, 2018 to allow for at least one year of follow up for all other patients.

BHCHP is a federally qualified health center that sees over 11,000 patients annually at over 80 sites in health centers, shelters and soup kitchens, a respite care unit and on street teams in the Boston metropolitan area.^8^ Self-reported homelessness is required to initially receive care at BHCHP; however, patients will often transition in and out of homelessness while still recieving care at BHCHP. In response to the high prevalence of HCV in its patient population,^9^ BHCHP established a dedicated HCV treatment team in January 2014 that provides comprehensive HCV management including treatment with DAAs.^10^

### Study Design

We linked the BHCHP cohort with the Massachusetts Department of Public Health (MDPH) Registry of Vital Records and Statistics (RVRS) death occurrence files that spanned January 1, 2003 to December 31, 2018 using Match*Pro, Version 1.6.3, a probabilistic record linkage application.^11^ We used first name, last name, date of birth, and social security number (SSN) to link records. Two investigators independently reviewed each match that achieved a threshold score ≥ 0.65.^12^ The investigators accepted pairs as a true match if there was alignment on at least one of the following National Death Index criteria:^13^ (1) SSN; (2) first name, last name, month and year of birth (± 1 year); (3) first name, last name, month and day of birth. A third investigator adjudicated discrepancies.

We used the International Statistical Classification of Diseases and Related Health Problems, Tenth Revision (ICD-10) codes for the underlying cause of death to identify and categorize liver-related deaths. The specific ICD-10 codes were selected based on prior studies of liver-related mortality and are outlined in Supplementary Table 1.^5–7^

Mortality data for the reference population, Massachusetts adults (age ≥ 18 years), was obtained through the CDC Wide-ranging Online Data for Epidemiologic Research (WONDER) Underlying Cause of Death files from 2003 to 2018.^14^

### Statistical analysis

We assessed overall liver-related mortality by calculating the age-standardized liver-related mortality rate in the BHCHP cohort aggregated across all study years. We also calculated age-standardized liver-related mortality rates aggregated across all study years stratified by sex, race and ethnicity. We used direct standardization procedures with weights chosen according to the age breakdown in the general Massachusetts population.^15^ We compared these standardized rates to the rates observed in the general Massachusetts population by calculating standardized mortality rate ratios (SRRs), or the standardized mortality rate in the BHCHP cohort divided by the crude mortality rate in the general Massachusetts population.

We evaluated temporal trends in liver-related mortality by plotting the yearly age-standardized mortality rates in the BHCHP cohort and the yearly crude mortality rates observed in the Massachusetts adult population. We then used the Joinpoint Regression Program, Version 4.9.1.0, to calculate the annual percent change in mortality rates for each cohort.^16^ We did not include the mortality rate in 2003 (the first year of the study period) in temporal analyses due to insufficient person-years of observation in the BHCHP cohort which made mortality estimates unreliable for that year.

To assess cause-specific liver-related mortality, we first tabulated the specific causes of liver-related deaths aggregated across the study period. We then calculated sex-stratified, age-standardized cause-specific mortality rates in the BHCHP cohort and compared these to the general Massachusetts population using SRRs. We were unable to stratify cause-specific liver-related mortality rates by race and ethnicity because the CDC WONDER database suppressed data in several strata where few deaths occurred.

All analyses were conducted from January 1, 2022 to September 7, 2022 using Stata/BE 17.0 (StataCorp) and Microsoft Excel (Microsoft Corp). We followed the STrengthening the Reporting of OBservational studies in Epidemiology (STROBE) guidelines.^17^ The MassGeneral Brigham Institutional Review Board approved this study with the determination that it was exempt from full review because it involved the use of existing data that cannot be linked back to individual subjects (exemption 45 CFR 46.101(b)).

## Results

The BHCHP cohort included 60,092 homeless-experienced adults observed for 520,430 person-years. A total of 7,130 deaths occurred during the study period. Of these deaths, 652 were liver-related with a crude liver-related mortality rate of 125.3 deaths per 100,000 person-years. Characteristics of liver-related decedents, non-liver-related decedents, and non-decedents are shown in Table 1. Liver-related decedents were majority male (79.3%); 61.2% were White, 17.2% Black, and 13.3% Hispanic or Latinx. The mean age of death for liver-related decedents was 55.7 (± 8.3) years old compared to 53.4 (± 13.6) years old for non-liver-related decedents.

### Overall liver-related mortality

Aggregated across the study period, the age-standardized liver-related mortality rate was 3.5-fold higher (95% CI: 3.4, 3.5) than the Massachusetts population (Table 2). The age-standardized liver-related mortality rate was highest among White males (164.0 deaths per 100,000 person years), followed by White females (119.3 deaths per 100,000 person years) and Hispanic or Latinx males (76.5 deaths per 100,000 person years) (Fig. 1). White females demonstrated the greatest disparity relative to the Massachusetts population (SRR 5.9, 95% CI: 5.7, 6.0), followed by White males (SRR 3.9, 95% CI: 3.9, 4.0) and Hispanic or Latinx females (SRR 3.3, 95% CI: 3.0, 3.6) (Fig. 1).

From 2004 to 2018, age-standardized liver-related mortality in the BHCHP cohort decreased on average by 3.5% annually (95% CI: −6.6%, −0.3%) while liver-related mortality in the Massachusetts population increased on average by 1.2% annually (95% CI: 0.9%, 1.6%) (Fig. 2). The trajectories of these two trends were significantly different (p = 0.001). Liver-related mortality remained significantly higher in the BHCHP cohort than the Massachusetts population each year of the study period.

### Cause-specific liver-related mortality

Cirrhosis (n = 157, 24%), liver cancer (n = 148, 23%), alcohol-related liver disease (n = 140, 21%), and viral hepatitis (n = 99, 15%) were the leading causes of liver-related mortality (Table 3). Hepatitis C accounted for 88 of 99 deaths (88.9%) categorized as viral hepatitis. Liver cancer was the leading cause of liver-related mortality in males (28.6 deaths per 100,000 person-years), and cirrhosis was the leading cause in females (17.7 deaths per 100,000 person-years) (Table 2). Each of the cause-specific liver-related mortality rates in the BHCHP cohort were substantially higher than those observed in the Massachusetts population (Table 2). The largest cause-specific mortality disparity with the Massachusetts population was viral hepatitis mortality among females (SRR 9.6; 95% CI 8.8, 10.4) (Table 2).

## Discussion

In this large cohort of people experiencing homelessness, liver-related mortality was substantially higher than in the general population across the 16-year study period. As one of the largest studies of mortality among people experiencing homelessness to date, we were able to expand on previously described liver-related mortality rates in homeless-experienced adults^4,18^ and uncover temporal, cause-specific, and demographic subgroup trends. Major findings included a decline in liver-related mortality over the study period, a high burden of viral hepatitis and alcohol-related liver disease mortality, and differences in liver-related mortality by sex, race, and ethnicity.

The decline in liver-related mortality observed in the BHCHP cohort is likely multifactorial. One contributing factor may be related to innovations in liver disease management and care delivery within the BHCHP health care system. With the advent of direct acting antivirals (DAAs), BHCHP developed a targeted clinical approach to treating patients with HCV, with 95% of those starting DAA therapy completing treatment and 85% achieving sustained viral response.^10^ It is unclear to what degree housing may have contributed to the decline in liver-related mortality, as patients receiving care at BHCHP often transition in and out of housing, and housing status is not always reliably documented within the electronic health record. A recent study described a reduction in all-cause and liver-related mortality in a cohort of chronically homeless individuals with HCV at 2 years after receiving supportive housing; however, there were no differences at 5 years.^19^ Housing, at least in the short term, may have a stabilizing effect on liver disease, although more data is needed to determine its long-term effect on liver disease and other health related outcomes. This finding should be interpreted with caution, as the decline in liver-related mortality could be confounded by the competing risk of death with the rapid rise in drug overdose in homeless-experienced adults during the same timeframe.^1,20^

Viral hepatitis deaths, the majority of which were HCV-related, was 7-fold higher in the BHCHP cohort compared to the general population. The high prevalence of HCV in populations of people experiencing homelessness is reflective of the high rates of substance use.^21^ Reducing HCV-related mortality in this population likely requires a multimodal approach including substance use harm reduction, regular HCV screening, and pragmatic access to HCV treatment. Harm reduction strategies, including access to sterile injection and non-injection supplies through syringe service programs, reduce transmission of HCV.^22^ Additionally, regular HCV screening is recommended for individuals at high risk, including those who use drugs.^23^ Moreover, the treatment landscape for HCV changed dramatically with the advent of DAAs. Low barrier clinic models providing access to these novel agents have achieved high rates of sustained virologic response in homeless-experienced adults.^10,24^

Alcohol-related liver disease mortality was 4-fold higher in the BHCHP cohort compared to the general population. The prevalence of unhealthy alcohol use is well described in people experiencing homelessness,^25^ with alcohol-attributable mortality contributing to the mortality disparity between homeless and general populations.^26^ Although medications for alcohol use disorder (MAUD) reduce the incidence of alcohol-related liver disease and are associated with lower incidence of decompensation in patients with liver cirrhosis,^27^ it is estimated that less than 2% of all patients with an alcohol use disorder are treated with these evidence-based medications.^28^ Moreover, less than 2% of patients hopsitalized with complications of alcohol use disorder will recieve MAUD within 30 days of discharge.^29^ MAUD, including extended-release naltrexone, are safe and effective in reducing alcohol-related harm in homeless-experienced adults.^30^ Further efforts are needed to rapidly expand access to alcohol use disorder treatment given the disparate burden of unhealthy alcohol use and associated mortality in this patient population.

Our study found differences in mortality based on sex, race, and ethnicity. Males had greater liver-related mortality when compared to females -- a trend similar to that seen in the general population.^31^ However, homeless-experienced females had a higher liver-related mortality disparity than males when compared to the general population. This finding has not been described elsewhere and more investigation is needed to both replicate in other contexts and evaluate the complex interplay of sex- and gender-based differences in homelessness that might in-part account for this mortality disparity.

Though Black, American Indian, Alaskan Native, and Hispanic or Latinx individuals experience homelessness at higher rates than White persons in the US,^32^ White males and females had the highest liver-related mortality rates and the largest relative liver-related mortality disparities when compared to the general population in this cohort. These findings are consistent with other descriptions of the intersection between homelessness, race and ethnicity, and mortality in the US.^33^ This discrepancy is likely a reflection of structural violence and racism creating a lower threshold for individuals from racially minoritized communities to experience homelessness, while in contrast, physical, mental, and behavioral health conditions often contribute to homelessness among White persons.^34^

There are several limitations in our study. The cohort was defined as having a clinical encounter with BHCHP, which selects for individuals who accessed tailored homeless health care services in Boston, Massachusetts. The study findings may not be generalizable to populations of people experiencing homelessness seeking care in other geographic locations or to populations who do not engage with homeless-tailored health care. In addition, as people often cycle in and out of homelessness, the contribution of an individual’s experience with homelessness to mortality was unknown. Limitations are also inherent in relying on ICD-10 codes to classify causes of death, including misclassification and diagnostic ambiguity. In cases in which cirrhosis, liver failure, and liver cancer were coded as the primary cause of death, it is unclear what role hepatitis C, alcohol, or other clinical etiologies of liver disease contributed to mortality. There were no deaths in our cohort catergorized as non-alcohol related fatty liver disease (NAFLD) or non-alcohol associated steatohepatitis (NASH) despite rising mortality in the US population in recent years.^7^ This is unexpected as homeless-experienced adults have a similar prevalence of obesity and metabolic risk factors as the general population.^35^ While the reason or this finding is unclear, it may be a limitation of using ICD-10 codes for classification.

## Conclusion

Though liver-related mortality rates decreased over time in this large cohort homeless-experienced adults, the rates remained substantially higher than in the general population across the study period. Viral hepatitis and alcohol-related liver disease deaths were notable contributors to liver-related mortality in this cohort. Mortality patterns based on sex, race, and ethnicity demonstrated that White females had the largest liver-related mortality disparity when compared to the general population. Interventions targeting the unique needs of people experiencing homelessness, including those that aim to prevent, identify, and treat HCV and unhealthy alcohol use, are needed to reduce liver-related mortality disparities in this high risk population.

## Figures and Tables

**Table 1:** Characteristics of decedents at the Boston Health Care for the Homeless Program from 2003–2018

	Liver-related decedents (n = 652)	Non-liver-related decedents (n = 6,478)	Non-decedents (n = 52,962)
Age at enrollment, mean (SD)	49.6 (8.06)	47.7 (12.85)	39.4 (12.80)
Sex, n (%)			
Male	517 (79.29)	5,021 (77.28)	32,555 (61.47)
Female	135 (20.7)	1,476 (22.72)	20,407 (38.53)
Race/ethnicity, n (%)			
White	399 (61.20)	3,800 (58.48)	21,978 (41.50)
Black	112 (17.18)	1,337 (20.58)	14,428 (27.24)
Latinx	87 (13.34)	628 (9.66)	10,063 (19.00)
Asian or Pacific Islander	2 (0.31)	38 (0.58)	708 (1.34)
American Indian or Alaskan Native	2 (0.31)	28 (0.43)	283 (0.53)
Other/more than one race/unknown	50 (7.67)	667 (10.26)	5,502 (10.39)
Age at death, mean (SD)	55.7 (8.29)	53.4 (13.64)	-
Autopsy performed, n (%)	86 (13.19)	2,310 (35.55)	-

**Table 2:** Mortality in the BHCHP cohort compared to the Massachusetts adult population from 2003–2018

	Entire cohort		Male		Female	
Cause of death	Rate*	SRR** (95% CI)	Rate*	SRR** (95% CI)	Rate*	SRR** (95% CI)
All liver-related	99.76	3.46 (3.41, 3.51)	114.39	2.88 (2.82, 2.93)	70.98	3.77 (3.68, 3.86)
Cirrhosis	23.52	3.24 (3.15, 3.34)	26.73	2.85 (2.74, 2.95)	17.67	3.34 (3.18, 3.50)
Liver cancer	22.27	2.43 (2.37, 2.50)	28.57	2.15 (2.08, 2.22)	6.07	1.13 (1.07, 1.19)
Alcohol-related	20.56	4.43 (4.28, 4.59)	23.97	3.49 (3.35, 3.65)	14.11	5.39 (5.06, 5.74)
Viral hepatitis	15.88	7.24 (6.90, 7.61)	18.04	5.82 (5.48, 6.18)	13.04	9.56 (8.78, 10.40)
Other liver disease	17.97	3.26 (3.15, 3.37)	17.43	2.47 (2.37, 2.84)	19.73	4.79 (4.56, 5.05)

* sge-standardized mortality rate per 100,000 person-years

** standardized mortality rate ratio; calculated as the age-standardized mortality rate in the Boston Health Care for the Homeless cohort divided by the crude rate in the general Massachusetts population

**Table 3:** Causes of liver-related death among adults at the Boston Health Care for the Homeless Program

Cause of death	n	Crude rate per 100,000 person years
Cirrhosis	157	30.17
Liver cancer	148	28.44
Alcohol related liver disease	140	26.90
Viral hepatitis	99	19.02
*Hepatitis C virus*	*88*	*16.91*
*Other and unspecified viral hepatitis*	*11*	*2.11*
Other liver disease	108	20.75
*Liver failure*	*46*	*8.84*
*Hepatorenal syndrome*	*5*	*0.96*
*Inflammatory liver disease*	*3*	*0.58*
*Chronic hepatitis*	*2*	*0.38*
*Fatty change of liver*	*2*	*0.38*
*Autoimmune liver disease*	*1*	*0.19*
*Other specified diseases of liver*	*1*	*0.19*
*Liver disease, unspecified*	*48*	*9.22*
